# Supplementary material for: Effects of Environmental and Socioeconomic Inequalities on Health Outcomes: A Multi-Region Time-Series Study
Source: Int J Environ Res Public Health. 2022 Dec 9;19(24):16521. doi: 10.3390/ijerph192416521 (PMC9778807; doi:10.3390/ijerph192416521)
Supplement: Supplementary file 1 [file ijerph-19-16521-s001.zip › ijerph-2035968-supplementary.pdf]

Effects of Environmental and Socioeconomic Inequalities on Health Outcomes: A Multi-Region Time-Series Study

Iara da Silva [1,2,*], Caroline Fernanda Hei Wikuats [1,2], Elizabeth Mie Hashimoto [1] and Leila Droprinchinski Martins [1]

1 Graduate Program in Environmental Engineering, Campus Londrina, Federal University of Technology—Paraná, Av. Dos Pioneiros, 3131, Londrina 86036-370, Paraná, Brazil

2 Department of Atmospheric Sciences, Institute of Astronomy, Geophysics and Atmospheric Sciences, University of São Paulo, Rua do Matão, 1226, São Paulo 05508-090, São Paulo, Brazil

* Correspondence: iara@alunos.utfpr.edu.br

Supplementary material

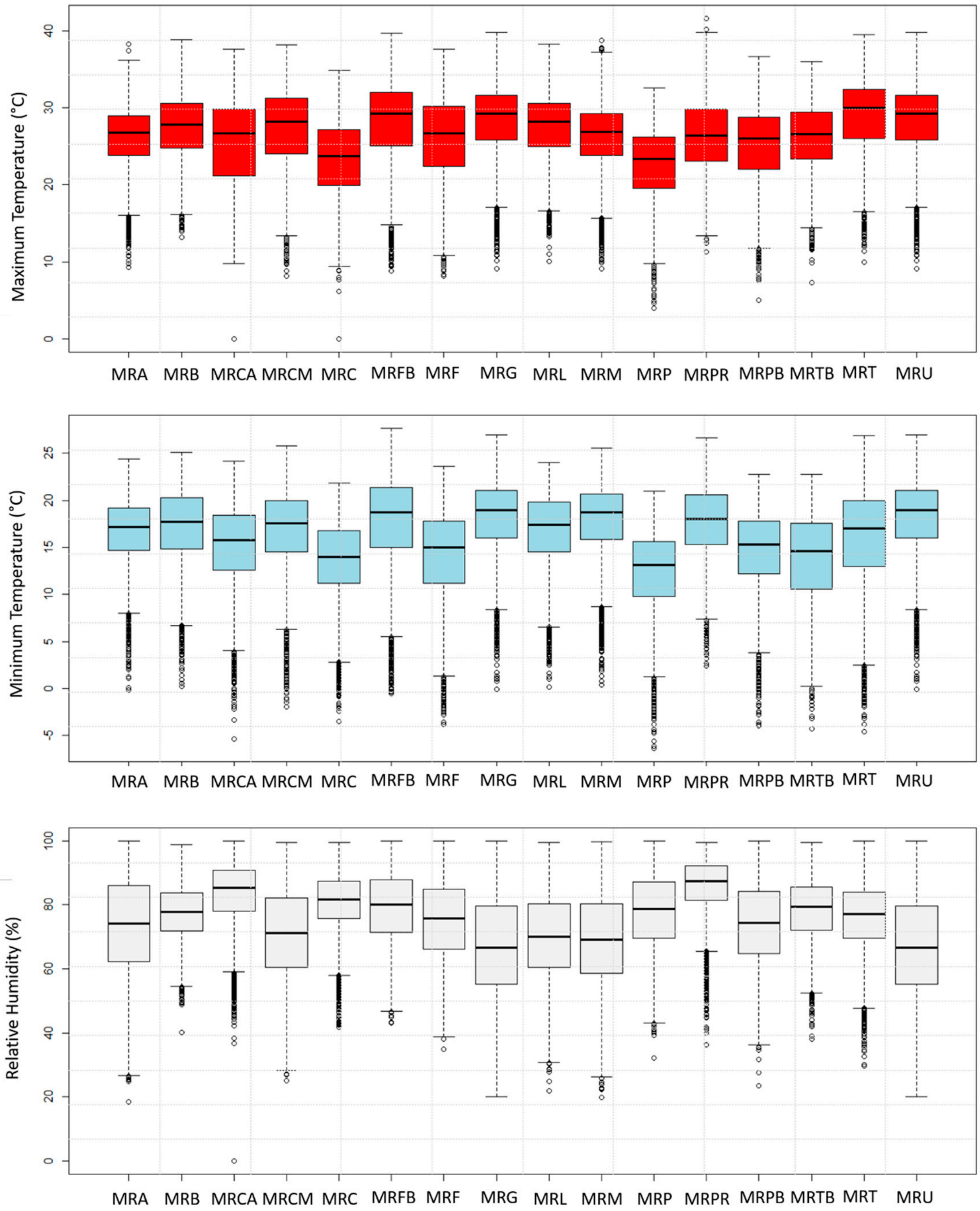

Figure S1. Boxplot of meteorological variables for regions in the period analyzed.

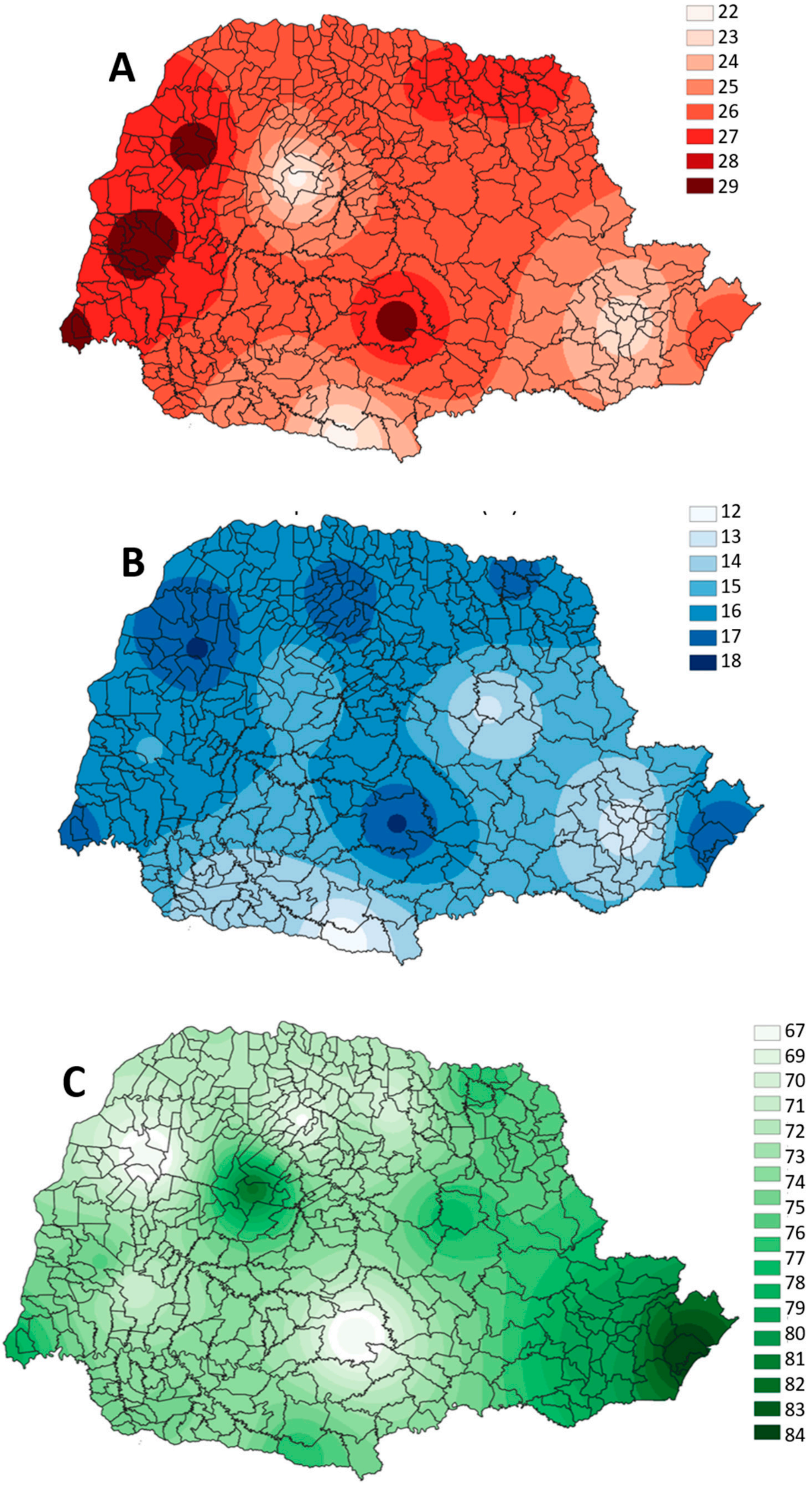

Figure S2. Average of (A) maximum and (B) minimum temperatures (°C). And (C) relative humidity (%) for the study period.

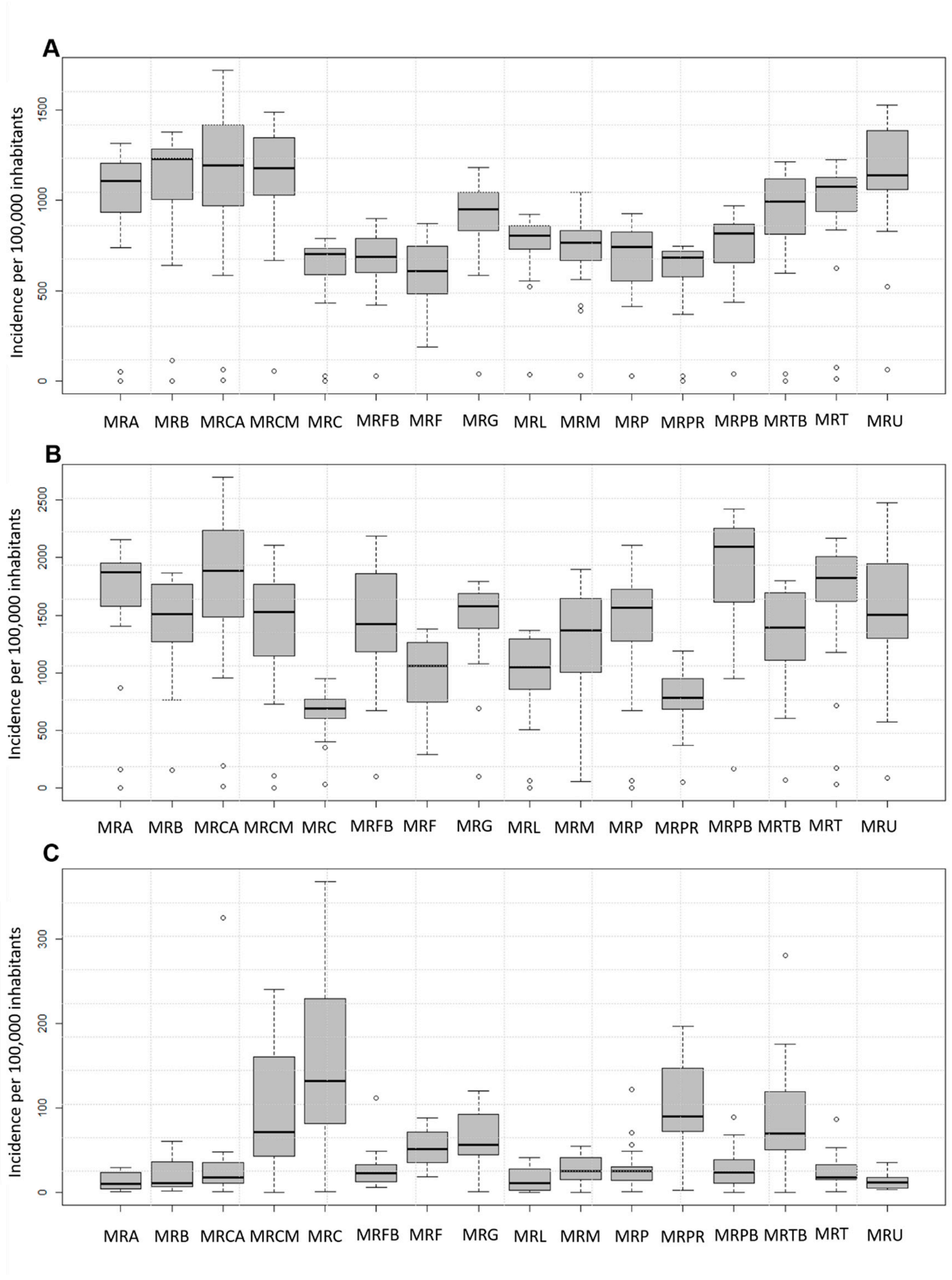

Figure S3. Boxplot of hospital admissions by diseases for regions in the period analyzed: (A) Cardiovascular, (B) Respiratory, and (C) Mental disorders.

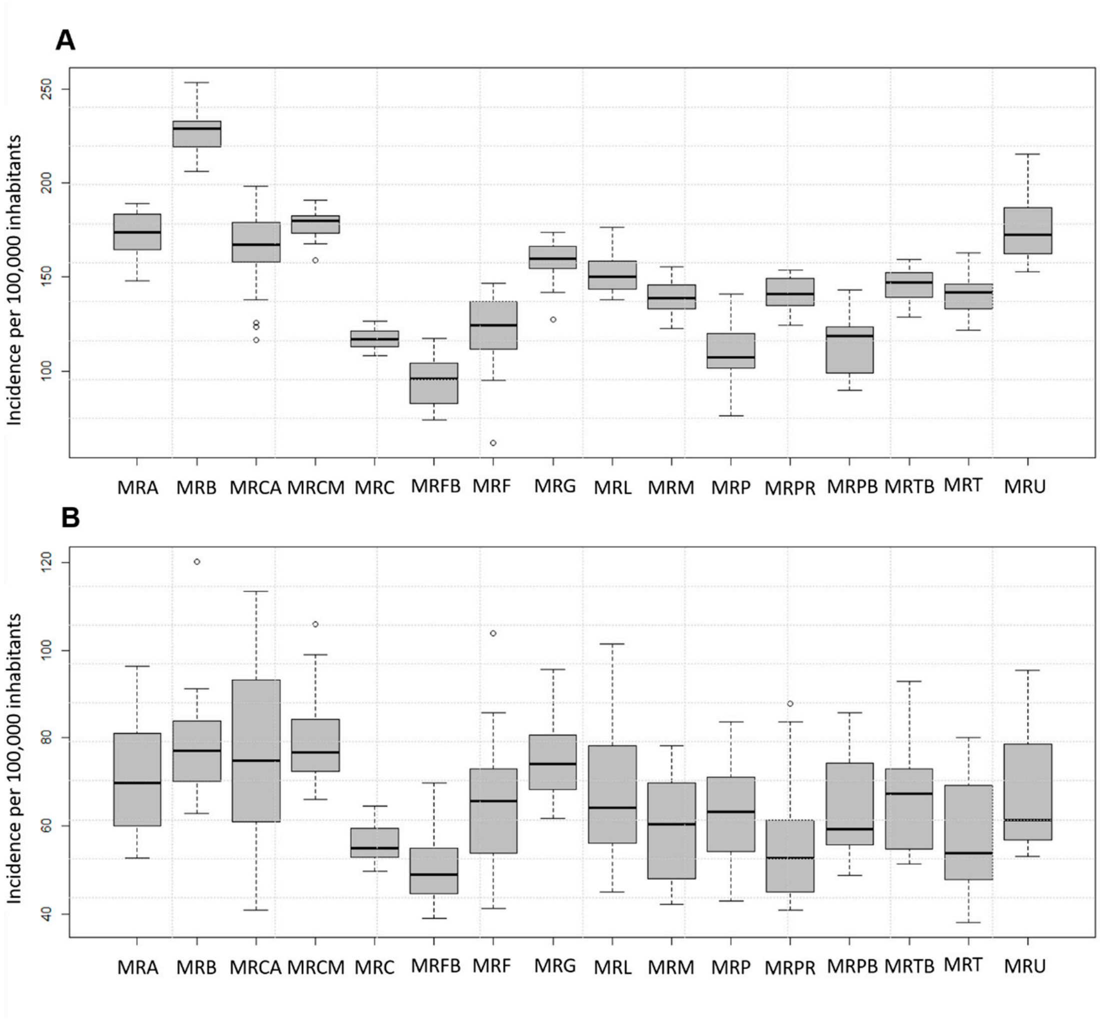

Figure S4. Boxplot of deaths by diseases for regions in the period analyzed: (A) Cardiovascular, (B) Respiratory.

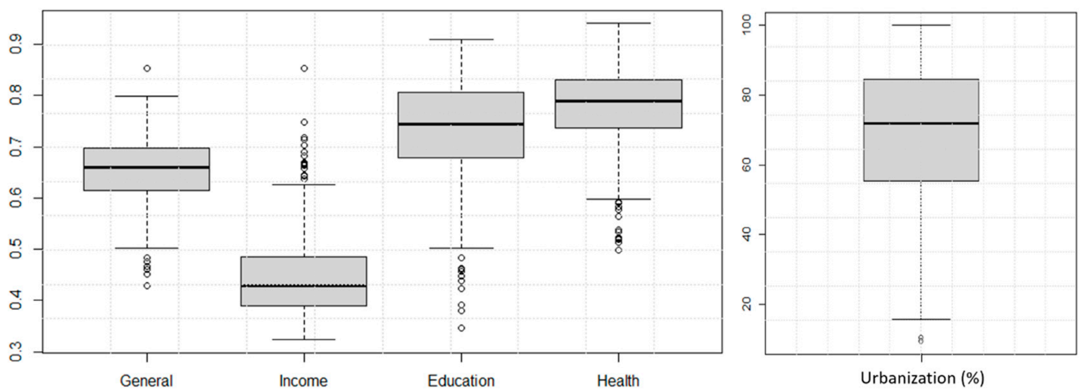

Figure S5. Average value (2010–2019) of the IPDM (Ipardes Municipal Development Index) – index that measures the performance of 399 municipalities in the state of Paraná, considering some dimensions: income, employment, production, health and education, and municipal urbanization rate.

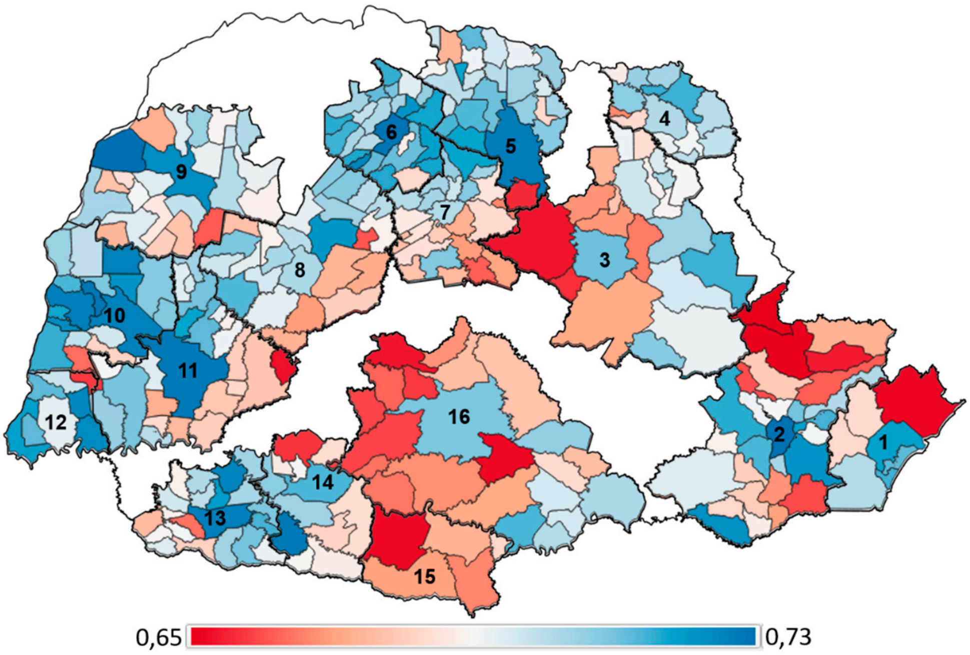

Figure S6. Municipal Human Development Index (MHDI) for the municipalities studied (2010 census) that composed the MR. MR's: Paranaguá (1), Curitiba (2), Telêmaco Borba (3), Bandeirantes (4), Londrina (5), Maringá (6), Apucarana (7), Campo Mourão (8), Umuarama (9), Toledo (10), Cascavel (11), Foz do Iguaçu (12), Francisco Beltrão (13), Pato Branco (14), Palmas (15), e Guarapuava (16). Bold contours indicate the municipalities in each MR.

The MHDI is a synthetic index that seeks to capture the socioeconomic conditions of the municipalities in the state of Paraná in its most significant dimensions: income (composed of income, employment, and agricultural production), education and health. It follows a similar line to the Human Development Index (HDI), prepared by the United Nations Development Programme (UNDP). The MHDI aims to provide the various spheres of government and civil society in general with an updated reading every year of relevant aspects of local development in the state. The index is built using different data sources of an administrative nature made available by public entities. The partial income index is constructed from data referring to work remuneration, formal employment, and agricultural production. In turn, the education index derives from information on early childhood education and basic education indicators, such as teachers with higher education, dropout rate, age-grade distortion rate, and Basic Education Development Index (IDEB). The health index comprises the percentage of prenatal consultations (more than six per child born alive), the share of deaths listed as ill-defined causes, and the ratio of deaths of children under five years of age from preventable causes among live births. Finally, the general MHDI index is calculated through the simple arithmetic mean of the partial indices mentioned above. Both the general and the partial indexes have values between 0 and 1, representing the minimum and maximum position of performance.

Next, the methodology for building the index is presented, separated by the three areas of development, namely: employment, income, and agricultural production; education; and health – each responsible for 33.33% (one-third) of the final index. The source is reference number 46, mentioned in the main manuscript.

1. Employment, Income, and Agricultural Production

1.1. Income

To obtain this component, the IPARDES Institute that calculates the index use information from annual data of the Work Ministry (in Portuguese Relação Anual de Informação Social do Ministério do Trabalho e Emprego (RAIS-TEM)). The growing rate of income and the absolute average income compose the variable income.

This indicator retains the greatest weight in the composition of the final index of employment, income, and agricultural production, corresponding to 42% of the total.

1.2. Employment

Employment comprises the formalization index, employment growth rate, and share of employment in the municipality in total employment in the state of Paraná. A score of 1 is assigned to municipalities with values greater than the 99th percentile, and a 0 score is assigned to those below the 1st percentile. This is done for the three factors.

1.3. Agricultural Production

To calculate the agricultural production sub-indicator, the gross value of municipal agricultural production and the gross value of production of animal origin were used, with data available at IBGE (https://www.ibge.gov.br/).

For the livestock sector, as the Gross Value of Production (GPV) is not available, the number of total carcasses of cattle, pigs, and poultry per municipality was estimated, and this amount was multiplied by the average price provided by IPEA (https://www.ipea.gov.br/portal/). Thus, considering the VBP, 1 is assigned to the municipality with the highest value.

2. Education

The Education dimension comprises indicators for kindergarten (20%), fundamental (69%) and secondary education (11%). The data source is the Ministry of Education, with indicators available at: http://portal.inep.gov.br/indicadores-educacionais and http://portalideb.inep.gov.br.

3. Health

The databases of the Secretariat of Health (SESA – Paraná) and the Population Projection calculated by IPARDES are used for health. The variables that compose the Health dimension are presented below.

3.1. Percentage of More Than Six Prenatal Consultations Per Child Born Alive

For the calculation of the maximum value, the percentage of 95% and the minimum of 0% were established. For the other municipalities, the values were scaled according to the percentage they have of the maximum score.

3.2. Percentage of Deaths From Ill-Defined Causes

The average percentage value equivalent to the 99th percentile in the 2008-2009-2010 triennium is used for the minimum level cut. For municipalities with a percentage greater than or equal to the minimum, a value of 1 was assigned, reflecting the worst situation. For the other municipalities, the values were obtained by the quotient between the percentage and the value of the 99th percentile. As the objective is to evaluate the best situation of the municipalities in this indicator, it was necessary to calculate its complement (100 minus the percentage of deaths due to ill-defined causes).

3.3. Percentage of Deaths of Children Under Five Years Old Due to Avoidable Causes by Live Birth

This indicator translates the ratio between the number of deaths of children under five from preventable causes per 100 live births in the same period. It follows the same criteria as indicated above in 3.2.

Table S1. Relative risk values with 95% confidence interval of hospitalization for cardiovascular diseases associated with explanatory variables for the groups and regions studied. The most significant lag are presented in parentheses.

Groups	MRA	MRB	MRCA	MRCM
GM1	-	-	-	TMAX - 1.0049 (1.0029-1.0069) (0)<br>TMAX - 1.0029 (1.0009-1.0049) (4)<br>TMAX - 1.0052 (1.0036-1.0068) (6)<br>MHDI - 0.8957 (0.8237-0.9740)
GM2	TMAX - 1.0167 (1.0083-1.0252) (0)<br>RH - 1.0022 (1.0008-1.0036) (0)<br>MHDI – 1.0002 (1.0001-1.0002)	TMIN - 1.0071 (1.0013-1.0130) (1)<br>MHDI – 1.0121 (0.9616-1.06528)	TMAX - 1.0057 (1.0001-1.0113) (0)<br>MHDI – 1.2087 (1.1444-1.2766)	TMAX - 1.0037 (1.0021-1.0053) (0)<br>TMAX - 1.0022 (1.0005-1.0039) (3)<br>TMAX - 1.0033 (1.0014-1.0051) (4)<br>MHDI – 0.8787 (0.8181-0.9437)
GM3	TMAX - 1.0100 (1.0013-1.0188) (0)<br>TMAX - 1.0129 (1.0042-1.0218) (7)<br>MHDI – 1.1548 (1.0337-1.2901)	-	TMAX - 1.0059 (1.0002-1.0116) (0)<br>MHDI – 1.3788 (1.2322-1.5427)	TMAX - 1.0041 (1.0009-1.0072) (4)<br>TMAX - 1.0086 (1.0061-1.0111) (6)<br>MHDI – 1.1102 (0.9734-1.2662)
GW1	TMIN - 1.0276 (1.0028-1.0530) (0)<br>MHDI – 1.0000 (1.0000-1.0000)	-	TMAX - 1.0104 (1.0059-1.0138) (0)<br>TMAX - 1.0048 (1.0014-1.0081) (3)<br>MHDI – 1.0419 (0.9965-1.0894)	TMAX - 1.0043 (1.0025-1.0062) (0)<br>TMAX - 1.0068 (1.0048-1.0087) (4)<br>TMAX - 1.0054 (1.0037-1.0071) (6)<br>MHDI – 0.7948 (0.7455-0.8474)
GW2	TMAX - 1.012 (1.0042-1.0195) (0)<br>MHDI – 1.3611 (1.2733-1.4549)	-	TMAX - 1.0083 (1.0054-1.0112) (0)<br>TMAX - 1.0037 (1.0002-1.0072) (3)<br>MHDI – 0.9173 (0.8677-0.9698)	TMAX - 1.0050 (1.0034-1.0067) (0)<br>TMAX - 1.0018 (1.0001-1.0034) (1)<br>MHDI – 0.8053 (0.7611-0.8520)
GW3	-	RH - 1.0040 (1.0004-1.0047) (2)<br>MHDI – 1.1999 (0.9580-1.5028)	-	TMAX - 1.0128 (1.0104-1.0153) (0)<br>TMAX - 1.0058 (1.0029-1.0087) (4)<br>TMAX - 1.0086 (1.0062-1.0109) (6)<br>MHDI – 1.1405 (1.0247-1.2695)

Groups	MRC	MRFB	MRF	MRG
GM1	RH - 1.0013 (1.0003-1.0023) (2)<br>RH - 1.0027 (1.0017-1.0037) (6)<br>MHDI – 0.8663 (0.8134-0.9226)	RH - 1.0036 (1.0017-1.0055) (2)<br>MHDI – 0.7891 (0.7160-0.8696)	-	TMAX - 1.0035 (1.0000-1.0071) (0)<br>MHDI – 1.0039 (0.9632-1.0464)
GM2	RH - 1.0020 (1.0011-1.0030) (6)	-	-	COM - 1.0054 (1.0030-1.0077) (0)

	MHDI – 0.9179 (0.8847-0.9524)			
GM3	RH - 1.0025 (1.0011-1.0040) (2)<br>MHDI – 0.8505 (0.8038-0.8998)	TMAX - 1.0193 (1.0078-1.0308) (0)<br>MHDI – 1.2722 (1.0492-1.5426)	TMAX - 1.0192 (1.0058-1.0329) (0)<br>MHDI – 1.2749 (1.0298-1.5785)	-
GW1	-	-	-	-
GW2	-	RH - 1.0022 (1.0009-1.0036) (2)<br>MHDI – 1.0747 (1.0056-1.1486)	-	TMIN - 1.0115 (1.0050-1.0180) (0)<br>MHDI – 1.2367 (1.1806-1.2954)
GW3	RH - 1.0025 (1.0011-1.0040) (2)<br>MHDI – 0.7285 (0.6653-0.7977)	TMAX - 1.0193 (1.0078-1.0308) (0)<br>MHDI – 0.8522 (0.7582-0.9579)	RH - 1.0095 (1.0052-1.0139) (3)<br>MHDI – 1.0623 (0.8733-1.2921)	TMAX - 1.0114 (10061-1.0167) (0)<br>TMAX - 1.0092 (1.0032-1.0152) (7)<br>MHDI – 1.2023 (1.0961-1.3187)
Groups	MRL	MRM	MRP	MRPR
GM1	-	TMAX - 1.0078 (1.0043-1.0113) (0)<br>MHDI – 0.9124 (0.8119-1.0253)	-	-
GM2	-	TMAX - 1.0062 (1.0002-1.0122) (0)<br>MHDI – 0.9508 (0.9029-1.0013)	-	TMAX - 1.0026 (1.0001-1.0088) (3)<br>MHDI – 1.5165 (1.2943-1.7769)
GM3	-	TMAX - 1.0011 (1.0010-1.015) (0)<br>TMAX - 1.0005 (1.0001-1.0042) (3)<br>MHDI – 1.1716 (1.0549-1.3011)	-	-
GW1	TMAX - 1.0410 (1.0032-1.0803) (1)<br>MHDI – 1.0000 (1.0000-1.0000)	TMAX - 1.0040 (1.0005-1.0076) (4)<br>TMAX - 1.0035 (1.0000-1.0069) (7)<br>MHDI – 1.5899 (1.5186-1.6646)	RH - 1.0048 (1.0014-1.0081) (1)<br>MHDI – 1.6713 (1.4188-1.9689)	-
GW2	-	TMAX - 1.0076 (1.0025-1.0039) (0)<br>TMAX - 1.0041 (1.0008-1.0074) (7)<br>MHDI – 0.8738 (0.8304-0.9194)	-	TMIN - 1.0009 (1.0007-1.0017) (5)<br>MHDI – 1.0931 (0.9828-1.2157)
GW3	-	TMAX - 1.0158 (1.0086-1.0230) (0)<br>RH - 1.0032 (1.0017-1.0012) (2)<br>MHDI – 0.8341 (0.7505-0.9271)	-	-
Groups	MRPB	MRTB	MRT	MRU
GM1	-	RH - 1.0017 (1.0000-1.0033) (2)<br>MHDI – 1.1863 (1.0899-1.2912)	TMAX - 1.0051 (1.0008-1.0094) (0)<br>TMAX - 1.0057 (1.0006-1.0107) (7)<br>MHDI – 0.8072 (0.7442-0.8755)	TMAX - 1.0048 (1.0002-1.0094) (0)<br>TMAX - 1.0062 (1.0009-1.0116) (3)<br>MHDI – 1.1100 (0.9722-1.2673)

GM2	-	RH - 1.0017 (1.0000-1.0032) (2)<br>MHDI – 1.0080 (0.9514-1.0679)	TMAX - 1.0094 (1.0037-1.0152) (0)<br>TMIN - 1.0087 (1.0033-1.0139) (5)<br>RH - 1.0035 (1.0017-1.0054) (5)<br>MHDI – 0.8109 (0.7478-0.8794)	-
GM3	-	-	TMAX - 1.0113 (1.0050-1.0177) (0)<br>TMAX - 1.0084 (1.0008-1.0161) (4)<br>MHDI – 2.0528 (1.8109-2.3268)	-
GW1	TMIN - 1.0098 (1.0011-1.0185) (1)<br>TMIN - 1.0100 (1.0013-1.0188) (4)<br>TMAX - 1.0109 (1.0028-1.0192) (7)<br>MHDI – 0.7063 (0.6301-0.7918)	TMIN - 1.0048 (1.0001-1.0096) (0)<br>TMAX - 1.0066 (1.0026-1.0106) (1)<br>RH - 1.0016 (1.0000-1.0030) (5)<br>MHDI – 1.0044 (0.9562-1.0550)	TMAX - 1.0104 (1.0064-1.0144) (0)<br>TMAX - 1.0054 (1.0007-1.0101) (4)<br>MHDI – 1.1282 (1.0460-1.2169)	-
GW2	RH - 1.0004 (1.0001-1.0018) (5)<br>RH - 1.0020 (1.0002-1.0038) (6)<br>MHDI – 0.6959 (0.6335-0.7645)	RH - 1.0015 (1.0001-1.0028) (2)<br>MHDI – 1.0883 (1.0020-1.8196)	TMAX - 1.0099 (1.0053-1.0147) (0)<br>TMAX - 1.0044 (1.0006-1.0081) (4)<br>RH - 1.0015 (1.0001-1.0029) (5)<br>MHDI – 1.2604 (1.1875-1.3377)	RH - 1.0013 (1.0002-1.0021) (2)<br>RH - 1.0011 (1.0002-1.0021) (6)<br>MHDI – 1.2333 (1.1181-1.3604)
GW3	TMAX - 1.0202 (1.0113-1.0291) (0)<br>TMAX - 1.0114 (1.0002-1.0228) (4)<br>MHDI – 1.4175 (1.1942-1.6828)	TMAX - 1.0151 (1.0095-1.0208) (0)<br>MHDI – 1.1319 (1.0250-1.2499)	TMAX - 1.0064 (1.0024-1.0155) (0)<br>TMIN - 1.0085 (1.0006-1.0164) (0)<br>TMIN - 1.0067 (1.0001-1.0108) (3)<br>RH - 1.0024 (1.0000-1.0047) (3)<br>MHDI – 1.1089 (1.0194-1.2062)	-

TMAX - Maximum temperature; TMIN - Minimum temperature; RH - Relative humidity; COM - Component.

Table S2. Relative risk values with 95% confidence interval of death for cardiovascular diseases associated with explanatory variables for the groups and regions studied. The most significant lag are presented in parentheses.

Groups	MRA	MRB	MRCA	MRCM
GM1	-	-	-	TMIN - 1.0135 (1.0011-1.0261) (4)<br>MHDI – 0.2806 (0.2116-0.3720)
GM3	-	TMAX - 1.0267 (1.0059-1.0478) (0)<br>RH - 1.0120 (1.0042-1.0199) (0)<br>MHDI – 1.0413 (0.8764-1.2373)	-	-
GW1	TMIN - 1.0276 (1.0028-1.0530) (0)<br>MHDI – 0.8571 (0.7340-1.0009)	-	-	-
GW2	-	TMIN - 1.0159 (1.0019-1.0302) (3)<br>MHDI – 1.0382 (0.9229-1.1678)	-	-

Groups	MRC	MRFB	MRF	MRG
GM1	-	TMIN - 1.0267 (1.0066-1.0472) (7)<br>MHDI – 1.3516 (1.0067-1.8147)	RH - 1.0089 (1.0029-1.0148) (0)<br>MHDI – 0.3755 (0.1749-0.8061)	TMIN – 1.0178 (1.0039-1.0318) (4)<br>MHDI – 1.0834 (0.9658-1.2153)
GM2	-	-	TMAX – 1.0134 (1.0042-1.0226) (1)<br>MHDI – 0.5043 (0.3277-0.7761)	-

Groups	MRL	MRM	MRP	MRPR
GM1	-	-	TMAX - 1.0378 (1.0052-1.0714) (5)<br>MHDI – 1.0491 (0.7691-1.4309)	TMIN - 1.0256 (1.0009-1.0510) (3)<br>MHDI – 1.1059 (0.9466-1.2921)
GM2	TMAX - 1.0203 (1.0095-1.0313) (0)<br>RH - 1.0031 (1.0008-1.0054) (0)<br>RH - 1.0022 (1.0009-1.0035) (2)<br>MHDI – 0.9121 (0.8691-0.9573)	-	RH - 1.0823 (1.0359-1.1308) (2)<br>MHDI – 1.1003 (0.7459-2.6336)	-
GM3	TMAX - 1.0260 (1.0149-1.0372) (0)<br>RH - 1.0031 (1.0007-1.0054) (0)<br>RH - 1.0022 (1.0008-1.0035) (2)<br>MHDI – 0.9771 (0.9083-1.0511)	-	-	-
GW1	-	-	RH - 1.0086 (1.0068-1.0183) (2)<br>MHDI – 0.2496 (0.2465-0.5203)	TMIN - 1.0359 (1.0069-1.0657) (5)<br>MHDI – 0.9087 (0.7605-1.0858)
GW3	-	-	-	RH - 1.0060 (1.0000-1.0121) (3)

MHDI – 1.3051 (1.1398-1.4944)

Groups	MRPB	MRTB	MRT	MRU
GM1	-	TMAX - 1.0124 (1.0002-1.0247) (1)<br>MHDI – 1.2805 (1.0786-1.5203)	-	-
GM2	-	-	-	COM - 1.0084 (1.0043-1.0125) (0)
GM3	-	-	RH - 1.0044 (1.0002-1.0085) (4)<br>MHDI – 1.0001 (1.0000-1.0001)	-
GW1	RH - 1.0285 (1.0051-1.0525) (3)<br>RH - 1.0371 (1.0150-1.0596) (4)<br>MHDI – 0.9991 (0.9987-0.9994)	TMIN - 1.0162 (1.0014-1.0311) (4)<br>MHDI – 0.9759 (0.7975-1.1942)	-	-
GW2	-	-	RH - 1.0040 (1.0005-1.0075) (2)<br>MHDI – 0.9999 (0.9999-0.9999)	RH - 1.0024 (1.0000-1.0047) (6)<br>MHDI – 0.7104 (0.4557-1.1073)

TMAX - Maximum temperature; TMIN - Minimum temperature; RH - Relative humidity; COM - Component.

Table S3. Relative risk values with 95% confidence interval of hospitalization for respiratory diseases associated with explanatory variables for the groups and regions studied. The most significant lag are presented in parentheses.

Groups	MRA	MRB	MRCA	MRCM
GM1	RH - 1.0018 (1.0002-1.0033) (3)<br>MHDI – 0.8737 (0.8006-0.9534)	-	-	TMAX - 1.0090 (1.0063-1.0118) (0)<br>TMAX - 1.0025 (1.0004-1.0046) (6)<br>RH - 1.0023 (1.0006-1.0039) (0)<br>MHDI – 0.9811 (0.8279-1.1625)
GM2	RH - 1.0017 (1.0000-1.0033) (3)<br>MHDI – 1.2137 (1.1069-1.3308)	-	-	-
GM3	RH - 1.0029 (1.0010-1.0048) (2)<br>RH - 1.0022 (1.0003-1.0041) (6)<br>MHDI – 1.2060 (1.0887-1.3360)	TMAX - 1.0269 (1.0103-1.0438) (0)<br>MHDI – 1.0569 (1.0087-1.2829)	-	TMAX - 1.0119 (1.0091-1.0147) (0)<br>TMAX - 1.0034 (1.0000-1.0068) (4)<br>MHDI – 0.8426 (0.6787-1.0460)
GW1	TMAX - 1.0410 (1.0032- 1.0803) (1)<br>MHDI – 0.0263 (0.00812-0.0845)	TMIN - 1.0136 (1.0037-1.0236) (0)<br>MHDI – 1.1849 (1.0195-1.3774)	-	TMAX - 1.0079 (1.0058-1.0102) (0)<br>RH - 1.0024 (1.0008-1.0039) (0)<br>MHDI – 1.7274 (1.5726-1.8974)
GW2	RH - 1.0012 (1.0000-1.0023) (5)<br>MHDI – 0.9073 (0.8535-0.9644)	-	TMIN - 1.0051 (1.0008-1.0094) (0)<br>MHDI – 1.0499 (1.0098-1.1154)	TMAX - 1.0096 (1.0077-1.0115) (0)<br>TMAX - 1.0034 (1.0014-1.0055) (4)<br>TMAX - 1.0025 (1.0007-1.0042) (6)<br>MHDI – 1.1839 (1.0917-1.2838)
GW3	RH - 1.0026 (1.0007-1.0045) (5)<br>MHDI – 1.0404 (1.0186-1.2531)	TMAX - 1.0286 (1.0125-1.0449) (0)<br>RH - 1.0081 (1.0021-1.0141) (0)<br>MHDI – 0.7028 (0.4361-1.1328)	TMIN - 1.0094 (1.0016-1.0172) (0)<br>MHDI – 1.0704 (1.0395-1.2049)	TMAX - 1.0042 (1.0013-1.0070) (0)<br>TMAX - 1.0043 (1.0010-1.0076) (4)<br>TMAX - 1.0031 (1.0002-1.0045) (6)<br>MHDI – 1.1572 (1.0227-1.3094)
**Groups**	**MRC**	**MRFB**	**MRF**	**MRG**
GM1	-	-	RH - 1.0025 (1.0000-1.0049) (5)<br>MHDI – 0.9745 (0.8592-1.1053)	-
GM2	-	-	RH - 1.0038 (1.0016-1.0059) (2)<br>RH - 1.0029 (1.0008-1.0051) (5)<br>MHDI – 0.8887 (0.81187-0.9728)	COM - 1.0025 (1.0000-1.0049) (0)
GM3	TMAX - 1.0144 (1.0077-1.0212) (0)<br>MHDI –0.8297 (0.7766-0.8865)	TMAX - 1.0135 (1.0029-1.0241) (0)<br>MHDI – 1.1544 (1.0297-1.2941)	-	-

GW1	-	TMAX - 1.0091 (1.0023-1.0161) (0)<br>RH - 1.0049 (1.0027-1.0072) (0)<br>MHDI – 1.0252 (1.0195-1.1023)	-	-
GW2	TMAX - 1.0059 (1.0019-1.0098) (0)<br>MHDI – 1.0658 (1.0233-1.1101)	-	-	-
GW3	-	TMAX - 1.0135 (1.0028-1.0257) (0)<br>MHDI – 1.1544 (1.0298-1.2941)	TMAX - 1.0139 (1.0026-1.0252) (4)<br>MHDI – 1.3072 (1.0947-1.5609)	-

Groups	MRL	MRM	MRP	MRPR
GM1	TMIN - 1.0133 (1.0062-1.0204) (4)<br>MHDI – 1.5882 (1.5064-1.6745)	-	-	TMAX - 1.0103 (1.0014-1.0193) (5)<br>MHDI – 1.2769 (1.1579-1.4082)
GM2	RH - 1.0013 (1.0000-1.0025) (2)<br>MHDI – 0.8602 (0.8079-0.9158)	-	-	-
GM3	TMAX - 1.0157 (1.0050-1.0265) (0)<br>MHDI – 1.1336 (1.0437-1.2313)	-	-	-
GW1	RH - 1.0017 (1.0004-1.0029) (5)<br>MHDI – 0.7745 (0.7282-0.8236)	-	-	TMAX - 1.0103 (1.0013-1.0194) (5)<br>MHDI – 1.2724 (1.1227-1.4421)
GW2	TMAX - 1.0059 (1.0010-1.0108) (0)<br>RH - 1.0011 (1.0000-1.0021) (2)<br>RH - 1.0012 (1.0001-1.0022) (5)<br>RH - 1.0016 (1.0006-1.0027) (6)<br>MHDI – 0.7886 (0.7475-0.8319)	-	-	-
GW3	RH - 1.0015 (1.0000-1.0031) (1)<br>MHDI – 2.3187 (2.1684-2.4795)	-	-	TMAX - 1.0196 (1.0011-1.0385) (0)<br>MHDI – 1.2563 (1.0429-1.5134)

Groups	MRPB	MRTB	MRT	MRU
GM1	-	-	-	RH - 1.0018 (1.0004-1.0032) (5)<br>MHDI – 1.0000 (1.0000-1.0000)
GM2	TMAX - 1.0154 (1.0061-1.0247) (0)<br>MHDI – 1.3314 (1.2005-1.4765)	-	TMAX - 1.0107 (1.0033-1.01823) (0)<br>RH - 1.0022 (1.0002-1.0042) (1)<br>RH - 1.0025 (1.0005-1.0045) (4)<br>MHDI – 0.9753 (0.8619-1.1035)	-

GM3	RH - 1.0029 (1.0001-1.0056) (4)<br>RH - 1.0039 (1.0011-1.0067) (5)<br>MHDI – 0.6688 (0.5725-0.7813)	-	-	-
GW2	TMIN - 1.0015 (1.0006-1.0023) (0)<br>MHDI – 1.0931 (1.0599-1.1961)	-	TMAX - 1.0098 (1.0038-1.0159) (0)<br>MHDI – 0.8222 (0.7718-0.8758)	-
GW3	TMAX - 1.0264 (1.0109-1.0422) (0)<br>MHDI – 1.3334 (1.1449-1.5529)	TMAX - 1.0121 (1.0051-1.0191) (0)<br>MHDI – 0.8435 (0.7600-0.9360)	TMAX - 1.0106 (1.0001-1.0211) (0)<br>MHDI – 1.3921 (1.2622-1.5354)	-

TMAX - Maximum temperature; TMIN - Minimum temperature; RH - Relative humidity; COM - Component.

Table S4. Relative risk values with 95% confidence interval of death for respiratory diseases associated with explanatory variables for the groups and regions studied. The most significant lag are presented in parentheses.

Groups	MRA	MRB	MRCA	MRCM
GM1	-	-	TMIN - 1.0268 (1.0009-1.0532) (7)<br>MHDI – 0.9352 (0.6428-1.3606)	TMIN - 1.0223 (1.0016-1.0435) (5)<br>MHDI – 0.1504 (0.0917-0.2469)
GW1	TMAX - 1.0410 (1.0032-1.0803) (1)<br>MHDI – 0.2629 (0.0817-0.8457)	RH - 1.0222 (1.0074-1.0373) (0)<br>RH - 1.0155 (1.0004-1.0309) (2)<br>RH - 1.0166 (1.0015-1.0319) (5)<br>MHDI – 0.5742 (0.3546-0.9295)	TMAX - 1.0316 (1.0056-1.0582) (2)<br>MHDI – 0.6366 (0.4219-0.9606)	-
GW2	-	-	-	TMIN - 1.0129 (1.0004-1.0254) (5)<br>MHDI – 0.3954 (0.2906-0.5381)
GW3	TMIN – 1.0266 (1.0057-1.0480) (1)<br>MHDI – 1.3713 (1.1419-2.9291)	-	-	-

Groups	MRC	MRFB	RMF	MRG
GM1	-	-	TMIN – 1.0370 (1.0137-1.0610) (3)<br>MHDI – 1.9281 (1.2524-1.4726)	-

Groups	MRL	MRM	MRP	MRPR
GM1	TMIN - 1.0049 (1.0036-1.0163) (0)<br>MHDI – 1.0055 (1.0191-1.0222)	TMIN - 1.0420 (1.0197-1.0649) (4)<br>MHDI – 0.9181 (0.7551-1.1164)	TMAX - 1.0534 (1.0009-1.1086) (6)<br>MHDI – 1.0823 (1.0594-1.9732)	-
GM2	-	-	-	-
GM3	-	-	TMIN - 1.0519 (1.0101-1.0956) (5)<br>MHDI – 1.7307 (1.1152-2.6858)	-
GW1	-	TMIN - 1.0742 (1.0306-1.1196) (0)<br>MHDI – 1.0086 (1.0061-1.0366)	-	-
GW2	TMIN - 1.0006 (1.0000-1.0015) (0)<br>MHDI – 1.0671 (1.0266-1.1262)	-	-	-

Groups	MRPB	MRTB	MRT	MRU
GM1	-	-	TMAX - 1.0225 (1.0017-1.0438) (7)<br>MHDI – 0.7971 (0.6519-0.9746)	-
GM2	-	-	-	COM - 1.0084 (1.0043-1.0125) (0)

GW1	-	RH - 1.0116 (1.0000-1.0232) (0)<br>MHDI – 1.7569 (1.3965-2.2104)	-	-
GW2	-	RH - 1.0064 (1.0006-1.0121) (0)<br>MHDI – 1.0001 (1.0001-1.0001)	TMAX - 1.0188 (1.0014-1.0365) (1)<br>MHDI – 0.7281 (0.3983-0.0.9133)	-
GW3	-	-	-	RH - 1.0052 (1.0008-1.0096) (4)<br>MHDI – 1.0002 (1.0001-1.0002)

TMAX - Maximum temperature; TMIN - Minimum temperature; RH - Relative humidity; COM - Component.

Table S5. Relative risk values with 95% confidence interval of hospitalization for mental illnesses and disorders associated with explanatory variables for the groups and regions studied. The most significant lag are presented in parentheses.

Groups	MRB	MRCA	MRCM
GM1	TMAX - 1.0108 (1.0103-1.0889) (0)<br>MHDI – 1.2719 (1.1952-1.3117)	-	TMAX – 1.0062 (1.0003-1.0159) (0)<br>TMIN – 1.0064 (1.0052-1.0163) (3)<br>MHDI – 1.4724 (1.0304-2.1039)
GW1	-	TMAX – 1.0442 (1.0007-1.0897) (0)<br>TMAX – 1.0705 (1.0209-1.1225) (4)<br>MHDI – 0.2573 (0.1348-0.4909)	TMAX - 1.0134 (1.0034-1.0292) (0)<br>MHDI – 0.9378 (0.5431-1.6195)
GW2	-	TMAX - 1.1651 (1.0607-1.2797) (5)<br>MHDI – 0.6751 (0.2091-0.2172)	TMAX - 1.0112 (1.0100-1.0289) (0)<br>TMAX - 1.0217 (1.0019-1.0496) (1)<br>TMAX - 1.0252 (1.0125-1.0552) (2)<br>TMAX - 1.0213 (1.0112-1.0480) (3)<br>MHDI – 1.1839 (1.0917-1.2838)

Groups	MRC	MRFB	RMF
GM1	TMIN - 1.0826 (1.0638-1.1017) (0)<br>TMIN - 1.0134 (1.0025-10245) (5)<br>TMIN - 1.0142 (1.0036-1.0249) (6)<br>MHDI – 0.5705 (0.4795-0.6788)	TMIN - 1.0013 (1.0011-1.0070) (0)<br>MHDI – 1.0004 (1.0002-1.0006)	-
GM2	TMIN - 1.1019 (1.0682-1.1367) (0)<br>TMIN - 1.0293 (1.0064-1.0528) (2)<br>TMIN - 1.0240 (1.0007-1.0479) (5)<br>TMIN - 1.0324 (1.00915-1.0561) (6)<br>MHDI – 0.5156 (0.4054-0.6555)	-	TMAX - 1.0446 (1.0079-1.0825) (2)<br>MHDI – 0.9211 (0.3511-2.4165)
GM3	TMIN - 1.0866 (1.0149-1.1633) (0)<br>TMIN - 1.1073 (1.0062-1.2186) (4)<br>MHDI – 1.1479 (1.8937-1.9582)	-	-
GW1	TMIN - 1.0541 (1.0404-1.0680) (0)<br>TMIN - 1.0156 (1.0047-1.0266) (2)<br>MHDI – 0.8097 (0.7161-0.9155)	-	TMAX - 1.0619 (1.0169-1.1089) (0)<br>RH - 1.0253 (1.0115-1.0394) (0)<br>MHDI – 1.1631 (1.0774-1.7481)
GW2	TMIN - 1.1194 (1.0892-1.1505) (0)<br>TMIN - 1.0239 (1.0014-1.0468) (5)<br>MHDI – 0.4352 (0.3398-0.5575)	-	-
GW3	TMIN - 1.0248 (1.0123-1.0964) (0)<br>MHDI – 1.8476 (1.0912-3.7441)	-	-

Groups	RMG	MRL	MRM
GM1	-	TMIN - 1.1331 (1.0399-1.2347) (0)<br>TMIN - 1.0858 (1.0283-1.1465) (5)<br>TMIN - 1.0705 (1.0124-1.1318) (6)<br>MHDI – 1.0747 (1.0459-1.5483)	TMIN - 1.1060 (1.0388-1.1775) (0)<br>MHDI – 1.4217 (1.3575-1.6149)
GM2	-	-	TMIN - 1.0655 (1.0034-1.1314) (0)<br>MHDI – 1.1439 (1.0781-1.9076)
GW1	-	TMIN - 1.0697 (1.0174-1.1246) (0)<br>MHDI – 1.4241 (1.0019-1.7012)	TMIN - 1.034 (1.0053-1.0639) (0)<br>MHDI – 2.4199 (1.6039-3.6509)

GW2	TMAX - 1.0457 (1.0138-1.0787) (5)<br>MHDI – 2.2524 (1.0491-4.8362)	-	-

Groups	MRPR	MRTB
GM1	TMIN - 1.0241 (1.0056-1.0428) (0)<br>MHDI – 1.2563 (1.0429-1.5134)	RH - 1.0163 (1.0063-1.0264) (0)<br>RH - 1.0198 (1.0096-1.0302) (2)<br>RH - 1.0149 (1.0045-1.0247) (4)<br>MHDI – 2.1434 (1.1186-4.1068)
GM2	-	TMAX - 1.0744 (1.0061-1.1474) (0)<br>TMIN - 1.0459 (1.0137-1.0790) (3)<br>RH - 1.0197 (1.0094-1.0301) (2)<br>RH - 1.0156 (1.0051-1.0261) (4)<br>MHDI – 2.1109 (1.1037-4.0371)
GW1	TMIN - 1.0504 (1.0009-1.1025) (0)<br>TMIN - 1.0511 (1.0022-1.1024) (5)<br>MHDI – 0.6217 (0.3909-0.9886)	TMIN - 1.0481 (1.0162-1.0811) (5)<br>MHDI – 2.9775 (1.4448-6.1361)
GW2	-	TMIN - 1.0864 (1.0301-1.1457) (0)<br>MHDI – 1.3464 (0.6492-1.8489)

TMAX - Maximum temperature; TMIN - Minimum temperature; RH - Relative Humidity.

Cardiovascular diseases						Respiratory diseases				
**Apucarana**										
Group	Selected variables	AIC values				Selected variables	AIC values			
		PO	BN	ZAP	ZANBI		PO	BN	ZAP	ZANBI
GM1	RH	15699.18	15674.73	17050.27	17051.68	RH	13903.86	13898.21	14994.3	14996.31
GM2	TMAX+TMIN+RH	21944.92	21663.0	24541.99	23533.13	TMAX+TMIN+RH	20918.53	20579.33	60094.6	22243.95
GM3	TMAX	10857.06	10849.3	11570.01	11569.31	RH	12305.98	11488.46	12307.31	12308.73
GW1	TMIN	3604.705	3602.583	3614.711	3616.711	TMAX	1342.09	1344.09	1495.012	1497.011
GW2	TMAX+TMIN+RH	21491.53	21217.33	22015.13	21976.61	RH	18846.48	18756.46	20744.31	20713.84
GW3	TMAXTMIN+RH	11332.18	11326.89	12253.26	12178.61	RH	11782.6	11765.32	12563.9	12565.9
**Bandeirantes**										
Group	Selected variables	AIC values				Selected variables	AIC values			
		PO	BN	ZAP	ZANBI		PO	BN	ZAP	ZANBI
GM1	TMIN	10933.92	10935.92	8786.432	8812.159	TMIN	14214.34	14204.11	15796.28	15796.73
GM2	TMIN	20512.5	20481.71	23382.89	23376.3	TMIN	17685.17	17658.16	19932.98	19931.38
GM3	RH	12026.65	12025.75	13143.05	13145.05	TMAX+TMIN+RH	10041.09	10036.27	10891.12	10887.22
GW1	TMIN	16626.97	16618.0	18894.13	18895.16	TMIN	15232.73	15195.9	17028.89	17021.04
GW2	RH	20582.75	20545.23	23471.16	23457.41	TMIN	17970.52	17935.93	20168.63	20154.58
GW3	RH	5511.684	5513.684	3429.884	3431.884	TMAX+TMIN+RH	10657.51	10643.89	11561.13	11552.02
**Cascavel**										
Group	Selected variables	AIC values				Selected variables	AIC values			
		PO	BN	ZAP	ZANBI		PO	BN	ZAP	ZANBI
GM1	TMIN	19103.14	19070.97	21651.04	21652.62	TMIN	18564.05	18504.42	20871.41	20856.27
GM2	COM	23415.81	23368.37	26908.51	26900.96	COM	23055.4	26275.86	26304.63	22960.91
GM3	TMAX	12516.63	12512.18	13638.61	13639.68	COM	13588.03	14830.67	14829.54	13577.77
GW1	TMAX	21783.02	21561.75	24939.05	24812.44	TMIN	19741.84	19657.96	22318.5	22284.72
GW2	TMAX	23577.72	23443.7	26977.91	26965.23	TMIN	22465.77	22369.87	25779.82	25736.32
GW3	RH	13909.16	13895.32	15191.61	15200.89	TMIN	13434.52	13414.99	14651.61	14652.68

Group	Selected variables	AIC values				Selected variables	AIC values			
		PO	BN	ZAP	ZANBI		PO	BN	ZAP	ZANBI
GM1	TMAX+TMIN+RH	22156.22	24958.1	25775.12	25755.26	TMAX+TMIN+RH	18669.08	18666.21	21600.05	21598.43
GM2	TMAX+TMIN+RH	32028.03	30973.69	30949.16	30878.11	TMIN	9215.204	9217.204	9354.872	9354.926
GM3	TMAX+TMIN+RH	15435.53	15436.96	17424.19	17424.94	TMAX+TMIN+RH	14701.35	14695.12	16442.47	16440.65

GW1	TMAX+TMIN+RH	23129.41	23040.49	26989.4	26942.46	TMAX+TMIN+RH	20596.52	20543.08	23873.5	23833.05
GW2	TMAX+TMIN+RH	31505.48	30620.92	30661.47	30615.79	TMAX+TMIN+RH	23397.74	23303.58	27221.09	27202.45
GW3	TMAX	16581.91	16581.89	18841.12	18840.49	TMAX+TMIN+RH	14757.29	14756.55	16528.85	16530.29
Curitiba										
Group	Selected variables	AIC values				Selected variables	AIC values			
		PO	BN	ZAP	ZANBI		PO	BN	ZAP	ZANBI
GM1	RH	38518.1	36846.32	41870.91	40956.02	TMIN	27582.62	27476.12	32029.13	31988.3
GM2	RH	41525.73	39178.15	44808.98	43342.15	TMIN	20055.44	20051.14	20133.68	20132.48
GM3	RH	24106.36	24037.07	27642.72	27638.45	TMAX+TMIN+RH	20376.75	20349.68	23285.13	23277.16
GW1	TMAX+TMIN+RH	38745.86	37022.43	41906.05	40941.78	TMIN	28716.94	28441.8	33070.1	32916.76
GW2	TMIN	26380.45	26366.94	26413.9	26409.0	TMAX+TMIN+RH	39833.67	31751.07	37648.53	36532.6
GW3	RH	24106.36	24037.07	27642.72	27638.45	TMIN	22300.75	25295.31	25452.84	25458.32
Francisco Beltrão										
Group	Selected variables	AIC values				Selected variables	AIC values			
		PO	BN	ZAP	ZANBI		PO	BN	ZAP	ZANBI
GM1	RH	15186.76	15167.93	16945.88	16944.64	TMIN	16542.85	16528.96	18718.07	18719.34
GM2	RH	19935.39	19900.79	22629.01	22629.31	TMIN	22421.51	22374.14	25899.39	25880.31
GM3	TMAX+TMIN+RH	12238.85	12236.85	13361.97	13363.97	TMAX+TMIN+RH	12539.34	12536.85	13767.04	13767.76
GW1	RH	17418.87	17277.54	19785.37	19710.94	TMAX+TMIN+RH	18334.83	18308.72	21097.57	21093.13
GW2	RH	20557.24	20513.98	23441.37	23437.11	TMIN	22421.51	22374.14	25899.39	25880.31
GW3	TMAX+TMIN+RH	12238.85	12236.85	13361.97	13363.97	TMAX+TMIN+RH	12539.34	12555.99	13767.04	13767.76
Foz do Iguaçu										
Group	Selected variables	AIC values				Selected variables	AIC values			
		PO	BN	ZAP	ZANBI		PO	BN	ZAP	ZANBI
GM1	TMAX	13794.96	13780.78	14418.9	14417.79	RH	12130.99	12118.39	12713.65	12712.64
GM2	TMIN	15217.72	15196.56	15965.73	15960.82	TMAX+TMIN+RH	14254.49	14235.33	15038.58	15036.72
GM3	TMAX	5064.516	5060.801	5182.55	5182.7	TMIN	6890.752	6889.99	7083.937	7078.455
GW1	TMIN	15155.14	14967.61	15931.25	15894.32	TMAX+TMIN+RH	11528.31	11506.85	12108.19	12107.24
GW2	TMIN	14264.2	14229.91	14939.7	14932.86	TMAX	12878.27	12825.66	13408.91	13408.06

Group	Selected variables	AIC values				Selected variables	AIC values			
GW3	RH	6500.074	6496.23	6689.168	6684.461	TMAX	6811.003	6801.001	7013.205	7002.774
**Guarapuava**										
Group	Selected variables	AIC values				Selected variables	AIC values			
		PO	BN	ZAP	ZANBI		PO	BN	ZAP	ZANBI
GM1	TMAX	18709.03	18697.3	21334.38	21333.01	COM	17039.04	17032.85	19150.43	19151.86
GM2	COM	21849.62	21841.3	25240.83	25234.55	COM	21645.53	21615.84	24780.62	24803.81
GM3	RH	11538.69	11530.32	12530.36	12532.36	COM	14508.37	14706.75	13928.99	13828.35
GW1	RH	23064.04	22862.57	24830.28	23010.05	COM	18907.96	18861.8	21361.34	21365.34
GW2	COM	22956.31	22883.89	26423.89	26388.42	COM	23006.48	22882.14	26206.44	26131.58
GW3	TMAX	13887.81	13885.29	15320.63	15305.28	COM	13425.26	13416.8	14717.61	14715.72
**Londrina**										
Group	Selected variables	AIC values				Selected variables	AIC values			
		PO	BN	ZAP	ZANBI		PO	BN	ZAP	ZANBI
GM1	TMIN	24187.7	24077.82	41788.5	41464.08	TMIN	20712.68	20667.63	23728.12	23706.43
GM2	RH	28989.78	28574.92	47285.15	46755.27	TMAX+TMIN+RH	25588.42	25467.23	29440.99	29400.54
GM3	RH	10905.32	59835.48	23258.93	23255.65	TMAX+TMIN+RH	19745.33	18075.82	20166.15	20162.52
GW1	TMAX	1342.09	1344.09	1495.012	1497.011	TMAX+TMIN+RH	20599.22	20528.23	23543.54	23505.57
GW2	RH	28412.67	28028.93	46646.51	46128.43	TMAX+TMIN+RH	24636.88	24492.72	28155.94	28110.02
GW3	TMAX	13573.27	13575.27	27479.09	27461.94	TMAX+TMIN+RH	18809.61	18755.31	21046.24	21032.78
**Maringá**										
Group	Selected variables	AIC values				Selected variables	AIC values			
		PO	BN	ZAP	ZANBI		PO	BN	ZAP	ZANBI
GM1	TMAX	21741.84	21697.29	25003.37	24996.62	TMAX+TMIN+RH	21380.37	20385.37	24870.81	24849.61
GM2	TMAX+TMIN+RH	25978.08	24536.20	30451.33	30125.35	TMAX+TMIN+RH	24639.33	24547.24	28585.77	28558.53
GM3	TMAX+TMIN+RH	15369.47	15351.63	17070.72	17071.1	TMAX+TMIN+RH	16145.08	16123.23	18026.67	18016.29
GW1	TMAX+TMIN+RH	23089.01	25966.98	26719.27	26659.51	TMAX+TMIN+RH	21756.62	21643.46	25360.59	25298.56
GW2	TMAX+TMIN+RH	25761.3	25673.41	29915.41	29891.06	TMAX+TMIN+RH	23691.82	23598.58	27447.59	27412.89
GW3	TMAX+TMIN+RH	17305.48	17275.88	19441.62	19500.58	TMAX+TMIN+RH	16574.7	16548.15	18446.05	18459.13
**Palmas**										
Group	Selected variables	AIC values				Selected variables	AIC values			

		PO	BN	ZAP	ZANBI		PO	BN	ZAP	ZANBI
GM1	TMIN	7562.83	7562.537	8135.253	8137.246	TMAX	6380.737	6381.298	6822.785	6814.633
GM2	TMAX	3812.296	3813.709	3909.234	387945.1	TMIN	2326.078	2329.542	2332.508	3148.899
GM3	RH	3069.56	3071.56	3165.32	3305.9	TMAX	3353.962	3354.655	3452.65	3585.079
GW1	RH	8493.989	8490.58	9249.039	9247.691	TMIN	7371.234	7365.963	7955.075	7947.886
GW2	RH	9334.425	9327.894	10130.15	10129.52	TMIN	9543.843	9540.6	10334.38	10334.56
GW3	TMAX	3445.963	3446.938	3601.695	3623.075	TMIN	3626.261	3626.987	3842.02	3863.115

Paranaguá

Group	Selected variables	AIC values				Selected variables	AIC values			
		PO	BN	ZAP	ZANBI		PO	BN	ZAP	ZANBI
GM1	RH	8868.3	8870.233	6696.407	6693.517	TMAX+TMIN+RH	10543.6	10798.69	10800.7	10792.05
GM2	TMAX+TMIN+RH	19759.9	17880.64	20311.91	20308.74	TMIN	4871.838	4841.075	4870.825	4872.583
GM3	RH	6763.586	7240.535	7237.417	7239.394	TMIN	5470.539	5472.525	5754.558	5761.065
GW1	TMAX	15684.14	13917.04	15686.12	15685.33	TMAX+TMIN+RH	9864.754	9865.542	10790.06	10792.05
GW2	TMAX+TMIN+RH	16108.72	16105.82	18318.03	18313.28	TMIN	11700.31	11697.32	12975.44	12976.97
GW3	TMIN	7492.802	7487.594	7979.815	7980.716	TMAX+TMIN+RH	6831.952	6498.887	6946.979	6948.586

Pato Branco

Group	Selected variables	AIC values				Selected variables	AIC values			
		PO	BN	ZAP	ZANBI		PO	BN	ZAP	ZANBI
GM1	TMIN	12349.78	12295.48	13447.46	13449.98	TMIN	13433.04	13404.71	14723.42	14726.39
GM2	RH	16010.36	15972.81	17841.98	17846.22	TMAX+TMIN+RH	19546.6	19483.21	22027.99	22021.04
GM3	RH	6687.975	6667.569	7065.247	7067.537	RH	10052.75	9995.723	10735.37	10761.8
GW1	TMAX+TMIN+RH	14672.14	14246.4	15850.83	15679.22	TMIN	15372.7	15331.87	17169.63	17165.54
GW2	RH	16039.78	15988.04	17726.93	17727.08	TMIN	19129.07	19062.27	21451.42	21447.68
GW3	TMAX	8609.233	8577.537	9246.103	9221.089	TMAX+TMIN+RH	10392.99	10370.94	11188.32	11188.61

Telêmaco Borba

Group	Selected variables	AIC values				Selected variables	AIC values			
		PO	BN	ZAP	ZANBI		PO	BN	ZAP	ZANBI
GM1	RH	21679.32	21630.1	25015.14	25007.18	TMIN	18279.25	18264.54	20927.71	20928.63
GM2	RH	25808.74	25680.59	29567.76	29535.57	TMIN	22257.78	22220.48	25668.2	25660.88

Group	Selected variables	AIC values PO	BN	ZAP	ZANBI	Selected variables	AIC values PO	BN	ZAP	ZANBI
GM3	RH	13852	13842.54	15262.16	15263.53	TMIN	13752.13	13750.94	15287.15	15285.68
GW1	TMAX+TMIN+RH	22990.25	22939.78	26630.64	26625.64	TMIN	20872.45	20834.57	24015.6	24009.59
GW2	RH	26094.35	26012.7	30137.14	30111.13	TMIN	27877.23	23934.59	27981.31	27743.38
GW3	TMAX	14848.73	14847.62	16629.12	16631.12	TMAX+TMIN+RH	14037.01	14031.08	15524.82	15524.88
**Toledo**										
Group	Selected variables	AIC values				Selected variables	AIC values			
		PO	BN	ZAP	ZANBI		PO	BN	ZAP	ZANBI
GM1	TMAX	16280.05	16248.37	17943.91	17936.95	TMIN	15764.5	15748.49	17277.38	17277.2
GM2	TMAX+TMIN+RH	20513.74	20487.23	23020.11	23008.73	TMAX+TMIN+RH	20098.88	20076.73	22227.44	22217.44
GM3	TMAX	10960.62	10959.78	11767.73	11767.42	RH	11445.76	11444.92	12334.72	12336.72
GW1	TMAX	17700.77	17614.61	19746.34	19702.33	TMIN	17114.27	17075.54	18920.44	18910.44
GW2	TMAX+TMIN+RH	20484.11	20461.49	22869.77	22859.03	TMAX+TMIN+RH	18938.11	18901.41	20961.87	20944.11
GW3	TMAX+TMIN+RH	12707.49	12709.38	13828.34	13830.34	TMAX+TMIN+RH	11140.72	11136.56	11962.81	11964.81
**Umuarama**										
Group	Selected variables	AIC values				Selected variables	AIC values			
		PO	BN	ZAP	ZANBI		PO	BN	ZAP	ZANBI
GM1	TMAX	15509.14	15477.29	17191.5	17193.5	RH	14253.38	14249.93	15888.05	15886.14
GM2	COM	20282.11	20274.95	23165.59	23162.46	COM	17959.85	17937.64	20330.4	20318.21
GM3	RH	12208.95	12209.85	13197.53	13199.09	RH	11593.97	11594.02	12500.05	12502.05
GW1	TMAX	3860.145	3862.145	4201.866	4191.8	COM	1322.169	7161.356	16376.8	1256.153
GW2	RH	19726.18	19764.37	22387.75	22367.18	COM	17293.89	17278.02	19554.29	19551.68
GW3	RH	11989.46	11981.86	12986.26	12987.24	RH	9815.229	9816.738	10443.95	10445.95

TMAX - Maximum temperature; TMIN - Minimum temperature; RH - Relative humidity; COM - Component; PO - Poisson; BN - Negative binomial; ZAP - Poisson with zero adjustment; ZANBI - Negative binomial with zero adjustment.

Table S7. AIC values for selecting variables and models for hospitalizations for mental illnesses and disorders.

Mental illnesses and disorders					
Bandeirantes					
Group	Selected variables	AIC values			
		PO	BN	ZAP	ZANBI
GM1	TMAX	1604.219	1022.61	1005.392	1050.239
GM2	TMAX	198.578	141.375	171.725	224.113
GM3	-	-	-	-	-
GW1	RH	1174.982	769.343	1950.79	879.369
GW2	TMIN	89.744	89.044	154.056	148.442
GW3	-	-	-	-	-
Cascavel					
Group	Selected variables	AIC values			
		PO	BN	ZAP	ZANBI
GM1	TMAX	3334.171	2743.438	2927.319	3041.831
GM2	TMAX	846.964	701.787	808.824	816.265
GM3	TMAX	60.674	57.377	72.096	108.10
GW1	TMAX	2358.563	1666.335	1764.058	1824.606
GW2	TMAX	730.755	570.263	575.1	752.0
GW3	-	-	-	-	-
Campo Mourão					
Group	Selected variables	AIC values			
		PO	BN	ZAP	ZANBI
GM1	TMAX+TMIN+RH	9199.725	6591.542	6948.226	6849.778
GM2	TMIN	2305.304	1533.713	1553.6	1651.116
GM3	-	-	-	-	-
GW1	TMAX	8416.67	6389.468	6939.831	6912.51
GW2	TMAX	2024.902	1500.657	1576.312	1645.789
GW3	-	-	-	-	-
Curitiba					
Group	Selected variables	AIC values			
		PO	BN	ZAP	ZANBI
GM1	TMAX+TMIN+RH	69162.46	27786.78	67255.86	29951.18
GM2	TMAX+TMIN+RH	21250.99	10324.89	15333.66	10685.88
GM3	TMIN	1441.421	926.646	941.578	967.825
GW1	TMAX+TMIN+RH	41968.19	23448.52	37728.27	25906.1
GW2	TMAX+TMIN+RH	19489.25	9474.861	13758.18	10035.15
GW3	TMIN	970.285	778.373	1961.78	789.731
Francisco Beltrão					
Group	Selected variables	AIC values			
		PO	BN	ZAP	ZANBI
GM1	TMIN	3959.325	1955.379	2022.444	2014.577
GM2	-	-	-	-	-

GM3	-	-	-	-	-
GW1	TMIN	2256.56	1524.98	1598.35	1635.53
GW2	-	-	-	-	-
GW3	-	-	-	-	-

Foz do Iguaçu

Group	Selected variables	AIC values			
		PO	BN	ZAP	ZANBI
GM1	TMAX+TMIN+RH	4981.226	4934.202	5319.098	5316.578
GM2	TMAX	1549.396	1467.314	1791.895	1939.559
GM3	-	-	-	-	-
GW1	TMAX+TMIN+RH	2575.849	2288.803	9831.11	2421.641
GW2	-	-	-	-	-
GW3	-	-	-	-	-

Guarapuava

Group	Selected variables	AIC values			
		PO	BN	ZAP	ZANBI
GM1	RH	5920.046	5413.724	5956.077	5882.404
GM2	RH	1569.527	1561.399	1696.153	1719.295
GM3	-	-	-	-	-
GW1	RH	5246.02	5061.314	5537.385	5489.685
GW2	TMAX	1987.303	1968.874	2056.269	2720.604
GW3	-	-	-	-	-

Londrina

Group	Selected variables	AIC values			
		PO	BN	ZAP	ZANBI
GM1	TMAX+TMIN+RH	4121.629	2686.954	2836.001	2914.929
GM2	-	-	-	-	-
GM3	-	-	-	-	-
GW1	TMIN	3380.232	2015.811	67887.22	2145.195
GW2	-	-	-	-	-
GW3	-	-	-	-	-

Maringá

Group	Selected variables	AIC values			
		PO	BN	ZAP	ZANBI
GM1	TMAX+TMIN+RH	5147.468	3482.833	3488.576	10573.49
GM2	TMIN	2109.733	1274.976	1270.366	1340.601
GM3	-	-	-	-	-
GW1	TMIN	4128.362	3364.062	4142.329	3571.359
GW2	RH	1417.729	1109.639	1140.43	1131.676
GW3	-	-	-	-	-

Paranaguá

Group	Selected variables	AIC values

		PO	BN	ZAP	ZANBI
GM1	TMIN	6502.501	6498.887	6346.979	6948.586
GM2	-	-	-	-	-
GM3	-	-	-	-	-
GW1	TMIN	5865.63	3702.829	3927.659	3913.564
GW2	-	-	-	-	-
GW3	-	-	-	-	-
**Pato Branco**					
Group	Selected variables	AIC values			
		PO	BN	ZAP	ZANBI
GM1	TMIN	2058.508	1456.704	1859.954	1634.983
GM2	-	-	-	-	-
GM3	-	-	-	-	-
GW1	RH	1552.081	1308.569	1689.873	1650.682
GW2	-	-	-	-	-
GW3	-	-	-	-	-
**Telêmaco Borba**					
Group	Selected variables	AIC Model			
		PO	BN	ZAP	ZANBI
GM1	RH	6921.54	6729.617	8707.112	6784.491
GM2	TMAX+TMIN+RH	3869.975	1781.563	1834.96	1854.083
GM3	-	-	-	-	-
GW1	TMIN	9441.62	5058.284	6887.174	7411.693
GW2	TMIN	3843.314	1350.419	2174.048	1420.659
GW3	-	-	-	-	-

TMAX - Maximum temperature; TMIN - Minimum temperature; RH - Relative humidity. PO - Poisson; BN - Negative binomial; ZAP - Poisson with zero adjustment; ZANBI - Negative binomial with zero adjustment.

Table S8. AIC values for selecting variables and models for deaths from cardiovascular and respiratory diseases.

Cardiovascular diseases						Respiratory diseases				
Apucarana										
Group	Selected variables	AIC values				Selected variables	AIC values			
		PO	BN	ZAP	ZANBI		PO	BN	ZAP	ZANBI
GM1	RH	4660.806	4661.335	4674.76	4676.276	RH	1917.317	1919.317	2152.094	2154.095
GM2	TMIN	10067.52	10069.47	10087.64	10089.64	TMIN	5807.861	5809.549	5811.549	6218.307
GM3	TMIN	10067.52	10069.47	10087.64	10089.64	TMIN	5809.549	5811.549	6218.307	6220.287
GW1	TMIN	3602.705	3604.583	3614.711	3616.711	TMAX	1342.09	1344.09	1495.012	1497.011
GW2	TMAX	8407.623	8409.623	8434.824	8436.823	TMAX	4457.072	4892.71	4893.566	5047.243
GW3	TMIN	7023.385	7048.557	7050.561	7097.706	TMAX+TMIN+RH	4534.99	4550.938	4972.292	4974.292
Bandeirantes										
Group	Selected variables	AIC values				Selected variables	AIC values			
		PO	BN	ZAP	ZANBI		PO	BN	ZAP	ZANBI
GM1	TMIN	5924.614	5926.614	5931.525	5933.524	RH	2231.822	2233.822	2230.947	2243.142
GM2	TMIN	11781.58	11783.27	11800.98	11794.37	TMIN	5397.174	5399.174	5422.751	5424.751
GM3	TMAX+TMIN+RH	7894.774	7896.774	7923.108	7924.472	TMIN	4614.074	4616.074	4658.561	4643.834
GW1	TMAX	4335.421	4337.419	4340.522	4342.52	RH	1657.176	1659.176	2401.447	2046.941
GW2	TMIN	10213.38	10204.56	10219.73	10220.96	TMIN	4465.774	4467.774	4502.235	4504.373
GW3	TMIN	9417.034	9418.991	9449.071	9444.558	TMIN	4626.641	4625.001	4682.726	4684.766
Cascavel										
Group	Selected variables	AIC values				Selected variables	AIC values			
		PO	BN	ZAP	ZANBI		PO	BN	ZAP	ZANBI
GM1	TMIN	6085.815	6087.815	6092.279	6094.279	TMIN	2706.499	2708.499	2729.509	3220.056
GM2	TMIN	12321.41	12323.17	12363.1	12364.88	TMIN	7588.588	7590.223	7683.252	7677.092
GM3	TMAX	7782.719	7784.503	7872.875	7956.032	TMIN	5498.587	5500.571	5637.643	5639.118
GW1	TMAX	5254.076	5254.293	5256.293	5272.647	TMAX	2173.225	2175.225	2199.109	2201.109
GW2	TMAX	10762.15	10762.07	10789.94	10791.72	COM	5984.094	5886.094	6056.579	6055.265

Group	Selected variables	PO	BN	ZAP	ZANBI	Selected variables	PO	BN	ZAP	ZANBI
GW3	TMIN	8884.257	8885.146	9039.597	9031.193	TMIN	5246.446	5246.369	5418.149	5420.485
**Campo Mourão**										
Group	Selected variables	AIC values				Selected variables	AIC values			
		PO	BN	ZAP	ZANBI		PO	BN	ZAP	ZANBI
GM1	TMIN	8328.328	8330.328	8435.828	8437.616	TMIN	3932.077	3934.077	4020.51	4023.557
GM2	TMIN	15302.16	15304.16	15417.8	15419.8	TMIN	9215.204	9217.204	9354.872	9354.926
GM3	TMIN	10605.09	10607.09	10763.31	10762.45	TMIN	7188.451	7189.387	7314.674	7316.024
GW1	TMIN	6749.137	6751.137	6864.482	6866.389	TMIN	3174.693	3176.693	3239.135	3238.169
GW2	TMIN	13692.21	13694.21	13767.63	13769.41	TMIN	7947.196	7949.196	8091.129	8082.265
GW3	TMIN	11238.43	11240.1	11354.61	11353.65	TMAX/TMIN/RH	6562.972	6564.968	6757.052	6759.095
**Curitiba**										
Group	Selected variables	AIC values				Selected variables	AIC values			
		PO	BN	ZAP	ZANBI		PO	BN	ZAP	ZANBI
GM1	TMIN	20001.82	19994.07	20006.38	20008.39	TMIN	11602.12	11588.22	11667.79	11669
GM2	TMIN	27371.8	27370.26	27406.98	27404.45	TMIN	20055.44	20051.14	20133.68	20132.48
GM3	TMIN	20203.97	20205.03	20391.82	20393.77	TMIN	16093.62	16095.54	16302.35	16300.7
GW1	TMIN	17479.18	17480.64	17511.66	17513.67	TMIN	9590.31	9590.505	9669.988	9662.773
GW2	TMIN	26380.45	26366.94	26413.9	26409	TMIN	19496.8	19491.65	19655.98	19656.65
GW3	TMIN	24468.7	24464.98	24651.26	24651.37	TMAX+TMIN+RH	18284.44	18267.8	18545.56	18544.68
**Francisco Beltrão**										
Group	Selected variables	AIC values				Selected variables	AIC values			
		PO	BN	ZAP	ZANBI		PO	BN	ZAP	ZANBI
GM1	TMIN	3942.658	3944.658	3950.224	3953.039	TMIN	16542.85	16528.96	18718.07	18719.34
GM2	TMIN	9193.581	9193.066	9209.858	9211.858	TMAX+TMIN+RH	6683.675	6685.412	6723.424	6724.405
GM3	TMIN	5902.195	5903.826	5974.851	5976.927	TMIN	4665.291	72834.98	4721.916	4723.916
GW1	TMIN	3391.608	3393.155	3395.56	11530	TMIN	1620.226	1622.226	1632.274	1660.574
GW2	TMIN	8299.382	8301.382	8325.591	8327.567	TMIN	4658.975	4660.975	4704.599	4707.037

GW3	TMAX+TMIN+RH	7566.715	7568.715	7696.757	7698.799	TMAX+TMIN+RH	4315.681	4317.681	4419.76	4421.76
Foz do Iguaçu										
Group	Selected variables	AIC values				Selected variables	AIC values			
		PO	BN	ZAP	ZANBI		PO	BN	ZAP	ZANBI
GM1	RH	4446.538	4448.538	4803.846	4802.544	TMAX+TMIN+RH	2555.209	2557.209	2778.613	2780.476
GM2	TMAX	8903.794	8905.794	9222.661	9224.66	TMIN	5951.737	5953.737	6277.278	6279.26
GM3	TMIN	4901.198	4903.198	5239.682	5241.682	TMIN	3818.63	3820.63	4042.614	4034.759
GW1	TMAX	3694.776	3696.776	4028.796	4026.361	RH	1725.408	1727.408	1907.904	1909.895
GW2	TMIN	7205.143	7563.745	7565.745	7568.246	TMIN	4188.318	4190.318	4432.676	4434.676
GW3	RH	5815.521	5817.521	6098	6095.868	TMIN	3530.57	3532.57	3770.87	5403.574
Guarapuava										
Group	Selected variables	AIC values				Selected variables	AIC values			
		PO	BN	ZAP	ZANBI		PO	BN	ZAP	ZANBI
GM1	TMIN	7021.824	7023.824	7034.422	7036.422	TMIN	3611.72	3609.853	3616.006	3612.709
GM2	COM	13275.52	13177.52	13322.19	13324.16	COM	8732.9	8727.216	8777.847	8770.366
GM3	COM	8349.84	8341.105	8412.801	8413.746	TMIN	5323.096	5323.945	5406.724	5406.724
GW1	COM	6278.958	6278.726	6204.982	6306.959	COM	3244.776	3344.644	3356.784	3257.904
GW2	COM	12766.83	12668.1	12825.21	12827	COM	8394.117	8392.21	8462.887	8464.108
GW3	COM	10344.77	10346.77	10442.58	10444.58	COM	5781.017	5783.017	5900.087	5902.09
Londrina										
Group	Selected variables	AIC values				Selected variables	AIC values			
		PO	BN	ZAP	ZANBI		PO	BN	ZAP	ZANBI
GM1	TMIN	11722.49	11729.74	11725.28	11724.88	TMAX+TMIN+RH	20286.33	20295.59	20315.34	20317.34
GM2	TMAX+TMIN+RH	13148.28	13155.16	13207.48	13201.18	TMIN	20293.96	20195.59	20315.34	20317.34
GM3	TMAX+TMIN+RH	11527.08	11532.33	11717.94	11719.9	TMIN	9087.174	9084.537	9090.505	9092.44
GW1	RH	3672.607	3664.607	3676.521	3678.52	RH	17851.27	17852.88	17884.04	17886.01
GW2	TMIN	10842.04	10849.94	10938.08	10940.03	TMIN	17226.88	17136.36	17329.25	17331.25

Group	Selected variables	AIC values				Selected variables	AIC values			
GW3	TMAX+TMIN+RH	11811.35	11871.16	12106.35	12103.81	TMAX+TMIN+RH	17226.88	17236.36	17329.25	17331.25
**Maringá**										
Group	Selected variables	AIC values				Selected variables	AIC values			
		PO	BN	ZAP	ZANBI		PO	BN	ZAP	ZANBI
GM1	TMIN	8924.535	8926.535	8941.405	8943.407	TMIN	4166.878	4168.878	4183.914	4183.87
GM2	TMAX+TMIN+RH	16709.28	16711.28	16727.93	16729.74	TMIN	10180.35	10182.34	10214.67	10216.63
GM3	TMAX+TMIN+RH	12075.52	12077.46	12148.44	12150.44	TMIN	8580.721	8582.72	8714.582	8716.585
GW1	TMIN	6966.158	6968.143	6985.215	6986.307	TMAX+TMIN+RH	2683.547	2685.416	2707.256	2707.547
GW2	TMAX+TMIN+RH	14499.29	14496.97	14507.86	14508.65	TMIN	7822.948	7824.877	7872.441	7874.338
GW3	TMAX+TMIN+RH	13880.93	13882.93	13999.43	14001.43	RH	8613.995	8615.97	8783.031	8784.331
**Palmas**										
Group	Selected variables	AIC values				Selected variables	AIC values			
		PO	BN	ZAP	ZANBI		PO	BN	ZAP	ZANBI
GM1	TMAX	1862.199	1854.521	1859.678	2039.028	TMAX	887.8376	889.8376	891.4067	893.4067
GM2	RH	3812.296	3813.709	3809.234	3879.451	TMIN	2324.375	2324.942	2329.018	2331.018
GM3	TMIN	1718.278	1723.864	1729.825	1731.825	TMIN	1414.297	1416.297	1425.975	1427.975
GW1	RH	1957.346	1959.346	1954.501	1956.501	-	-	-	-	-
GW2	TMIN	3063.069	3063.586	3074.187	3075.165	TMIN	2031.401	2032.392	2051.575	2053.664
GW3	TMIN	2164.579	2166.579	2185.032	2187.038	TMIN	1381.73	1380.57	1382.019	1384.019
**Paranaguá**										
Group	Selected variables	AIC values				Selected variables	AIC values			
		PO	BN	ZAP	ZANBI		PO	BN	ZAP	ZANBI
GM1	TMIN	5096.088	5098.07	5118.437	5120.429	RH	2276.489	2278.489	2291.766	2293.765
GM2	TMIN	10300.97	10302.9	10306.24	10308.24	TMIN	4842.202	4842.052	4871.396	4873.075
GM3	TMIN	5555.016	5557.016	5578.785	5580.788	TMAX	2946.756	2945.438	3124.197	2991.12
GW1	TMIN	4055.306	4055.846	4066.831	4063.102	TMIN	1877.738	1878.526	1886.864	2442.572
GW2	TMIN	8390.454	8392.454	8408.934	8403.46	TMIN	4181.961	4183.942	4257.294	4183.565

Group	Selected variables	AIC values				Selected variables	AIC values			
GW3	RH	6616.023	6618.023	6633.849	6624.457	TMIN	3688.145	3689.93	3771.584	3771.679
**Pato Branco**										
Group	Selected variables	AIC values				Selected variables	AIC values			
		PO	BN	ZAP	ZANBI		PO	BN	ZAP	ZANBI
GM1	TMAX	2896.224	2879.406	2884.928	2895.647	TMIN	1128.621	1118.037	1131.132	1184.798
GM2	TMIN	6959.355	6912.581	6929.669	6922.153	TMIN	5021.661	5003.748	5033.572	5017.031
GM3	TMIN	4269.914	4246.57	4271.442	4276.08	TMAX	3558.26	3521.119	3576.538	3575.956
GW1	RH	3258.204	3224.791	3221.647	3224.23	TMIN	1579.552	1567.847	1569.561	1625.196
GW2	TMIN	6233.359	6190.531	6214.719	6203.415	TMIN	3914.683	3901.912	3936.883	3938.514
GW3	TMIN	5438.014	5408.034	5479.768	5481.769	RH	3249.882	3240.857	3269.485	3616.69
**Telêmaco Borba**										
Group	Selected variables	AIC values				Selected variables	AIC values			
		PO	BN	ZAP	ZANBI		PO	BN	ZAP	ZANBI
GM1	TMAX+TMIN+RH	8109.733	8111.733	8129.612	8131.61	TMAX+TMIN+RH	3800.087	3801.278	3828.216	3817.343
GM2	TMAX+TMIN+RH	14828.18	14829.71	14848.83	14850.77	TMIN	8676.779	8676.026	8778.967	8777.629
GM3	TMIN	9670.669	9711.117	9718.314	9719.848	TMAX+TMIN+RH	6239.881	6240.518	6334.03	6335.989
GW1	TMIN	6868.36	6880.114	6893.471	6882.112	TMAX+TMIN+RH	3317.963	3319.963	3342.402	3345.671
GW2	TMIN	13359.06	13358.62	13387.72	13389.72	TMAX+TMIN+RH	8321.228	8322.517	8432.705	8434.702
GW3	TMIN	10554.31	10556.13	10640.14	10638.01	TMIN	6340.659	6336.69	6482.271	6484.271
**Toledo**										
Group	Selected variables	AIC values				Selected variables	AIC values			
		PO	BN	ZAP	ZANBI		PO	BN	ZAP	ZANBI
GM1	TMIN	8111.696	8113.696	8128.777	8130.774	TMAX	3798.656	3799.792	3828.469	3804.182
GM2	TMIN	10780.25	10782.25	11170.3	11172.3	TMIN	6435.977	6437.977	6866.979	6868.957
GM3	TMAX+TMIN+RH	7070.929	7072.929	7560.641	7562.641	TMAX	4577.988	4579.988	5102.625	5098.737
GW1	TMIN	3649.877	3651.877	3957.071	3959.024	TMAX	1288.814	1290.814	1291.331	1488.452
GW2	TMAX+TMIN+RH	9041.821	9043.821	9427.759	9427.735	TMAX	3853.514	3855.514	4319.456	4321.438

GW3	TMIN	8483.956	8485.956	9035.042	9036.662	TMIN	3899.002	3901.002	4467.102	4469.102
Umuarama										
Group	Selected variables	AIC values				Selected variables	AIC values			
		PO	BN	ZAP	ZANBI		PO	BN	ZAP	ZANBI
GM1	TMIN	5409.849	5411.849	5675.926	5678.026	TMAX	2110.205	2112.937	2361.577	2363.791
GM2	COM	11204.34	11206.34	11515.87	11517.87	COM	6056.649	6058.649	6467.934	6466.405
GM3	TMIN	8068.677	8070.677	8514.971	8516.976	TMIN	4752.264	4754.264	5283.823	5285.663
GW1	TMAX	3860.145	3862.145	4189.644	4191.211	COM	1320.804	1323.732	1320.253	16376.8
GW2	RH	8927.688	8929.688	9300.364	9302.177	TMIN	3955.238	3952.315	4352.054	4353.829
GW3	TMIN	7918.749	7920.749	8394.036	8396.026	RH	3838.168	3840.168	4460.765	4462.788

TMAX - Maximum temperature; TMIN - Minimum temperature; RH - Relative humidity; COM - Component; PO - Poisson; BN - Negative binomial; ZAP - Poisson with zero adjustment; ZANBI - Negative binomial with zero adjustment.
